# Supplementary material for: RNA-binding activity of TRIM25 is mediated by its PRY/SPRY domain and is required for ubiquitination
Source: BMC Biol. 2017 Nov 8;15:105. doi: 10.1186/s12915-017-0444-9 (PMC5678581; doi:10.1186/s12915-017-0444-9)
Supplement: Additional file 1: Figure S1. — Thermal denaturation assays and size exclusion chromatography with multi-angle light scattering analyses show that ΔRBD deletion does not influence the overall shape and multimerization of purified His-TRIM25ΔRBD. Figure S2. Generation and characterization of TRIM25 KO HeLa cell line. Figure S3. T7-TRIM25 CLIP-seq and T7-TRIM25 RNA immunoprecipitation results. Figure S4. T7-TRIM25 CLIP-seq experiments are reproducible, based on three independent experiments. Figure S5. No correlation of TRIM25 CLIP-seq cluster intensities with transcript abundance in HeLa cells. Figure S6. Gene Ontology-term annotation of TRIM25-bound transcripts and the ab initio-derived TRIM25-binding binding sequences cluster in three motif clusters. Figure S7. TRIM25 does not influence RNA stability of selected targets. Figure S8. TRIM25 undergoes ubiquitination. (PDF 9873 kb) [file 12915_2017_444_MOESM1_ESM.pdf]

SUPPLEMENTARY INFORMATION

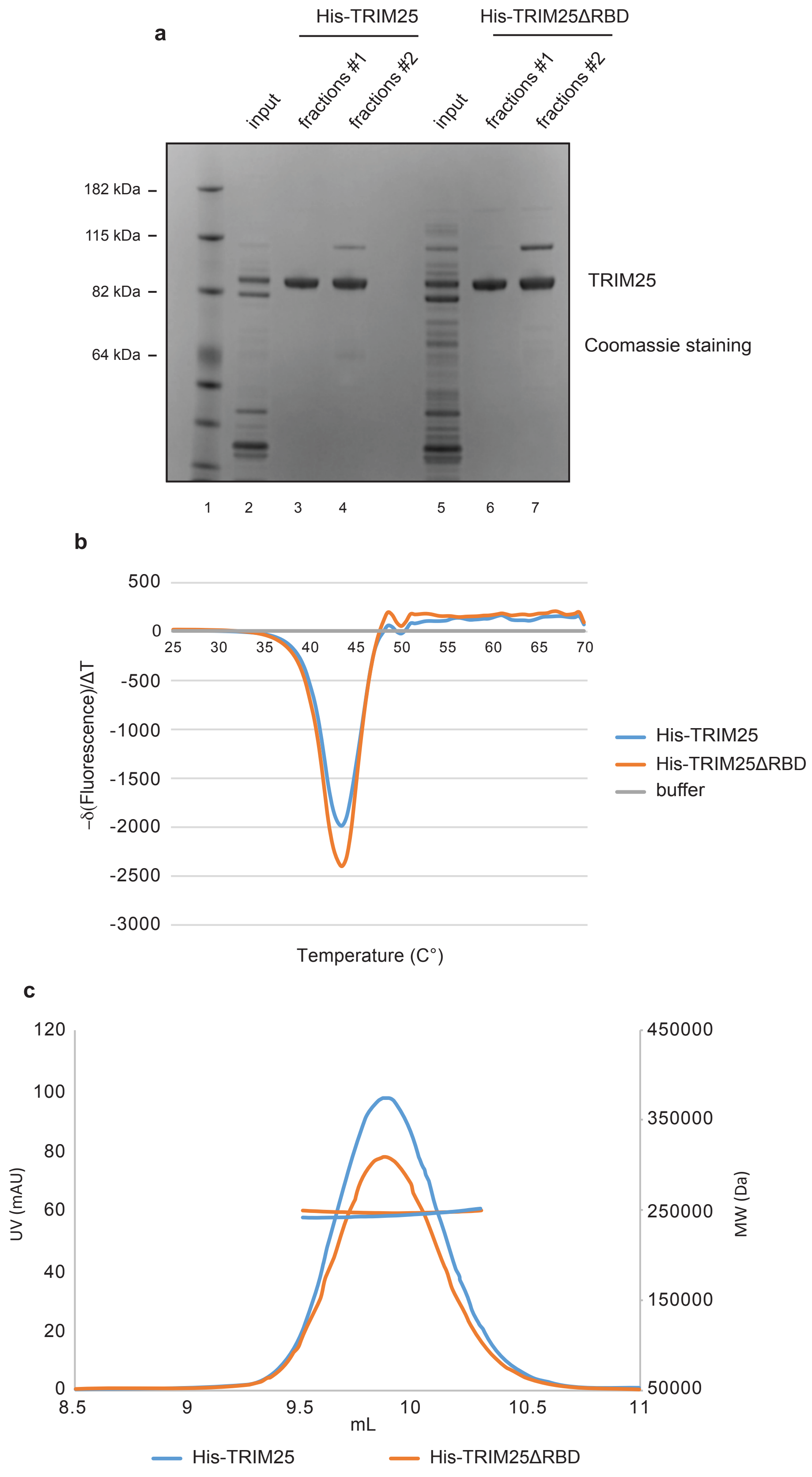

Supplementary Figure 1. Thermal denaturation assays and SEC-MALS analyses show that ΔRBD deletion does not influence the overall shape and multimerisation of purified His-TRIM25ΔRBD. (a) Coomassie-stained SDS-PAGE gel of purified proteins showing input to gel exclusion column (His-TRIM25 lane 2 and His-TRIM25ΔRBD lane 5) and two fractions pooled from gel exclusion fractions (His-TRIM25 lanes 3 and 4, His-TRIM25ΔRBD lanes 6 and 7) Lanes 1 and 5 show BenchMark Pre-Stained protein ladder. **(b)** Thermal denaturation assay of His-TRIM25 and His-TRIM25ΔRBD shows negative first derivatives of the raw curves. The peaks of the curves shows the T_m, 43°C ±0.3 (mean of three replicates ±S.D) for both proteins, indicating that they are both as stable. **(c)** SEC-MALS analysis of His-TRIM25 and His-TRIM25ΔRBD. Horizontal lines show the mass measurements. The corresponding theoretical masses are 263 ± 5.3 kDa (His-TRIM25) and 258 ± 5 kDa (His-TRIM25ΔRBD). Both are mainly tetrameric.

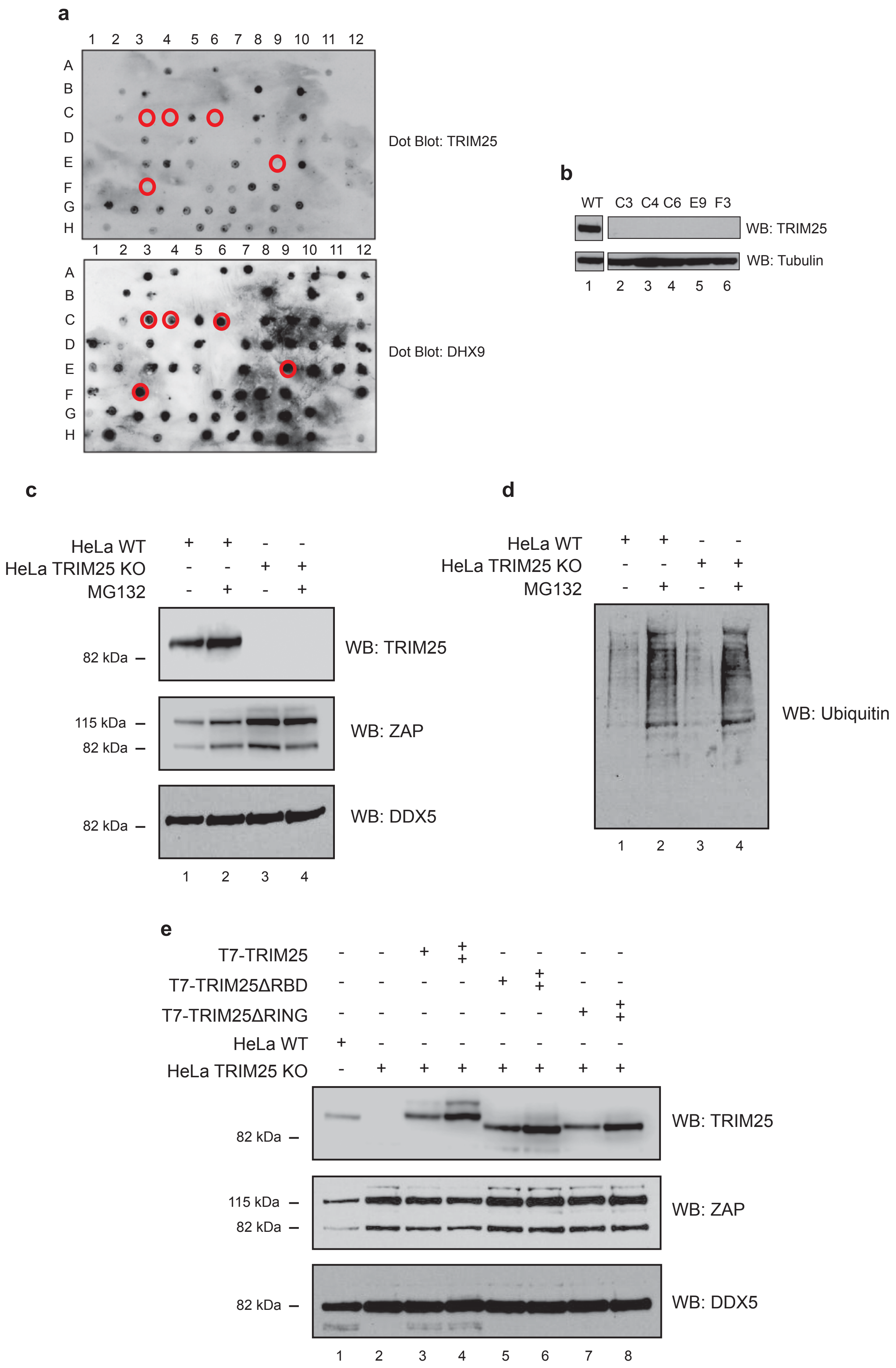
a
1 2 3 4 5 6 7 8 9 10 11 12
A B C D E F G H
Dot Blot: TRIM25
1 2 3 4 5 6 7 8 9 10 11 12
A B C D E F G H
Dot Blot: DHX9
b
WT C3 C4 C6 E9 F3
WB: TRIM25
WB: Tubulin
1 2 3 4 5 6
c
HeLa WT + + - -
HeLa TRIM25 KO - - + +
MG132 - + - +
82 kDa
WB: TRIM25
115 kDa
82 kDa
WB: ZAP
82 kDa
WB: DDX5
1 2 3 4
d
HeLa WT + + - -
HeLa TRIM25 KO - - + +
MG132 - + - +
WB: Ubiquitin
1 2 3 4
e
T7-TRIM25 - - + ++ - - - -
T7-TRIM25ΔRBD - - - - + ++ - -
T7-TRIM25ΔRING - - - - - - + ++
HeLa WT + - - - - - - -
HeLa TRIM25 KO - + + + + + + +
82 kDa
WB: TRIM25
115 kDa
82 kDa
WB: ZAP
82 kDa
WB: DDX5
1 2 3 4 5 6 7 8

Supplementary Figure 2. Generation and characterisation of TRIM25 KO HeLa cell line. (a) Dot blot of protein extracts from CRISPR/Cas9 treated clones. Levels of TRIM25 were analyzed by immunoblotting, with DHX9 as a loading control. Red circles signify clones that were picked for further analysis. **(b)** Western blot analysis of selected clones. Of note, HeLa cells have three copies of chromosome 17 were TRIM25 is placed, thus our KO cells are TRIM25$^{-/-/-}$. **(c)** Western blot analysis of wild type (WT) and TRIM25 KO HeLa cells shows upregulation of ZAP protein in the KO cells. Proteasome inhibitor MG132 treatment of wild type HeLa cells (Lane 2) increased levels of TRIM25 and ZAP as compared with untreated cells (Lane 1). ZAP levels were not altered by MG132 treatment in TRIM25 KO cells (Lane 4 vs Lane 3). DDX5 levels do not change and serve as a loading control. **(d)** The levels of ubqituitinated proteins are elevated in MG132-treated HeLa wild type and TRIM25 KO cells. Western analysis of ubiquitinated proteins upon MG132 treatment (Lanes 2 and 4) and untreated samples (Lanes 1 and 3). **(e)** Overexpression of T7-TRIM25 but not T7-TRIM25ΔRBD or T7-TRIM25ΔRING reduces the levels of ZAP. Western blot analysis on HeLa WT (Lane 1), HeLa TRIM25 KO cells (Lane 2), HeLa TRIM25 KO cells transfected with T7-TRIM25 (Lane 3; + - 50 ng, Lane 4 ++ - 100 ng per one 6 well), HeLa TRIM25 KO cells transfected with T7-TRIM25ΔRBD (Lane 5; + - 50 ng, Lane 6 ++ - 100 ng per one 6 well) and HeLa TRIM25 KO cells transfected with T7-TRIM25ΔRING (Lane 7; + - 25 ng, Lane 8 ++ - 50 ng per one 6 well). The proteins were analyzed by western blotting with anti-TRIM25, anti-ZAP and anti-DDX5 antibodies.

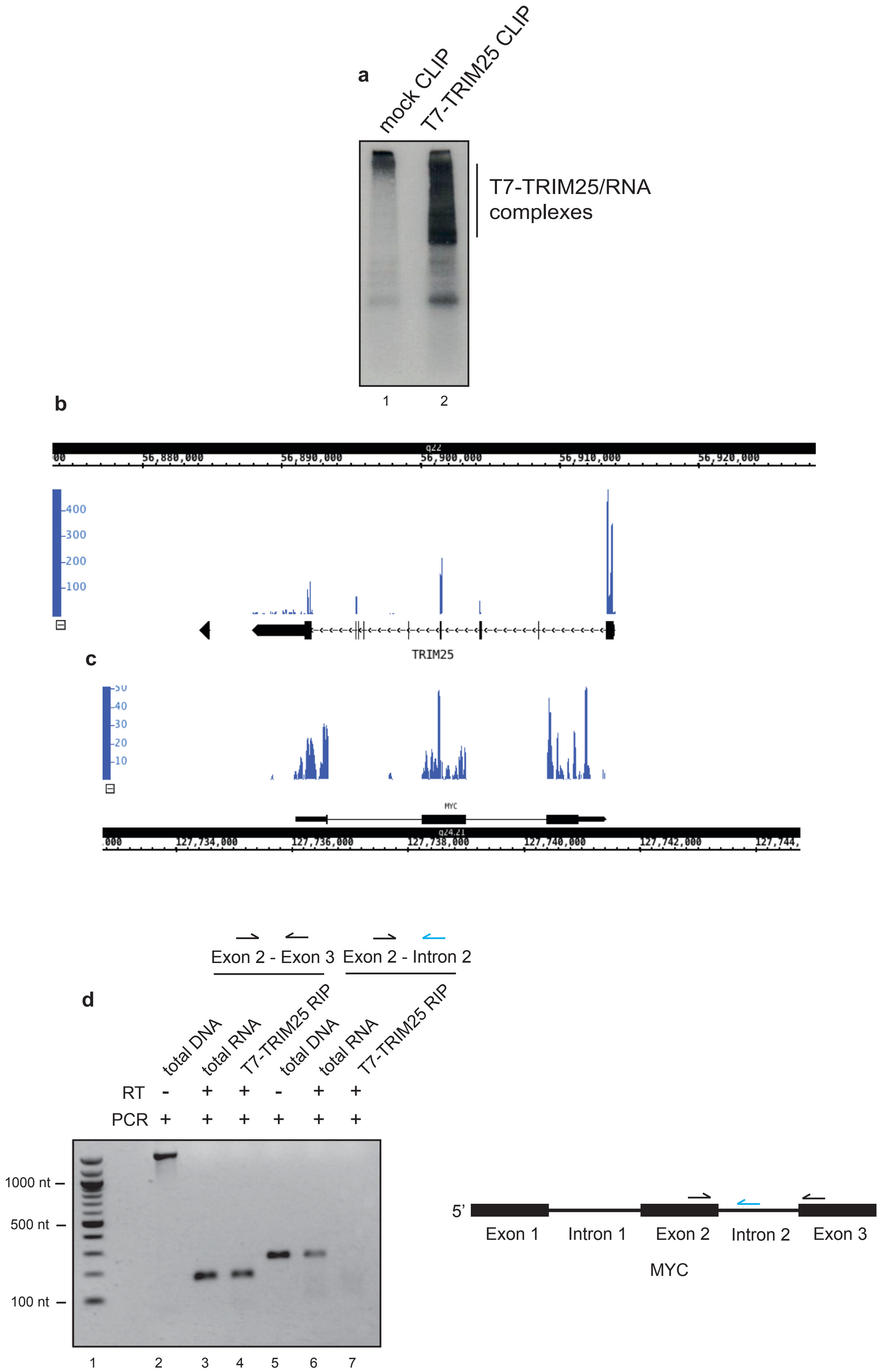

a
mock CLIP
T7-TRIM25 CLIP
T7-TRIM25/RNA complexes
1
2
b
q22
56,880,000
56,890,000
56,900,000
56,910,000
56,920,000
400
300
200
100
TRIM25
c
40
30
20
10
MYC
q24.21
127,734,000
127,736,000
127,738,000
127,740,000
127,742,000
Exon 2 - Exon 3
Exon 2 - Intron 2
d
total DNA
total RNA
T7-TRIM25 RIP
total DNA
total RNA
T7-TRIM25 RIP
RT - + + - + +
PCR + + + + + +
1000 nt
500 nt
100 nt
1 2 3 4 5 6 7
5'
Exon 1
Intron 1
Exon 2
Intron 2
Exon 3
MYC

Supplementary Figure 3. TRIM25 CLIP-seq (a) CLIP autoradiogram shows specific T7-TRIM25 RNA immunoprecipitation. An autoradiogram of CLIP of T7-TRIM25 in mock transfected (1) and T7-TRIM25 transfected (2) HeLa cells, showing T7-TRIM25/RNA complexes (RNase treated) specifically immunoprecipitated only in T7-TRIM25 transfected cells. **(b)** An example of TRIM25 CLIP-seq tag patterns over *TRIM25* mRNA (data visualized in Integrated Genome Browser). **(c)** An example of TRIM25 CLIP-seq tag patterns over *C-Myc* mRNA (data visualized in Integrated Genome Browser). **(d)** RNA immunoprecipitation (RIP) followed by targeted amplification of c-Myc transcript showing that TRIM25 predominantly interacts with mature mRNA. Agarose gel electrophoresis shows PCR on total genomic DNA isolated from HeLa cells using primers that span exon 2, intron 2 and exon 3 (Lane 1) and primers that span exon 2 and intron 2 (Lane 5). Total RNA was assayed by RT-PCR using primers that span exon 2, intron 2 and exon 3 (Lane 3) and primers that span exon 2 and intron 2 (Lane 6). RNA immunoprecipitation of T7-TRIM25 was assayed by RT-PCR using primers that span exon 2, intron 2 and exon 3 (Lane 4) and primers that span exon 2 and intron 2 (Lane 7).

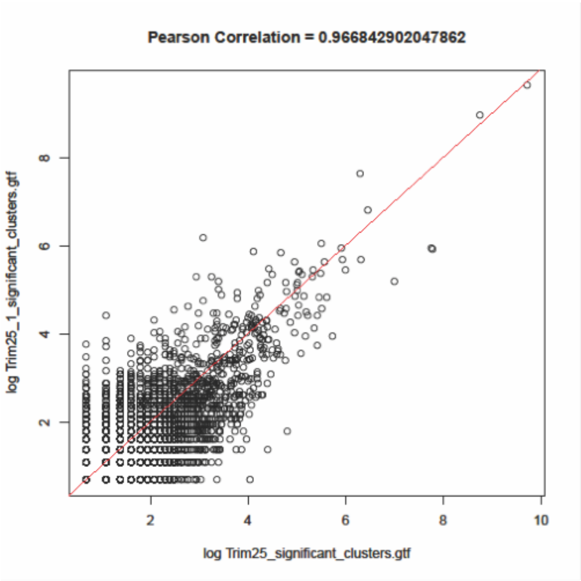
Pearson Correlation = 0.966842902047862
log Trim25_1_significant_clusters.gtf
log Trim25_significant_clusters.gtf

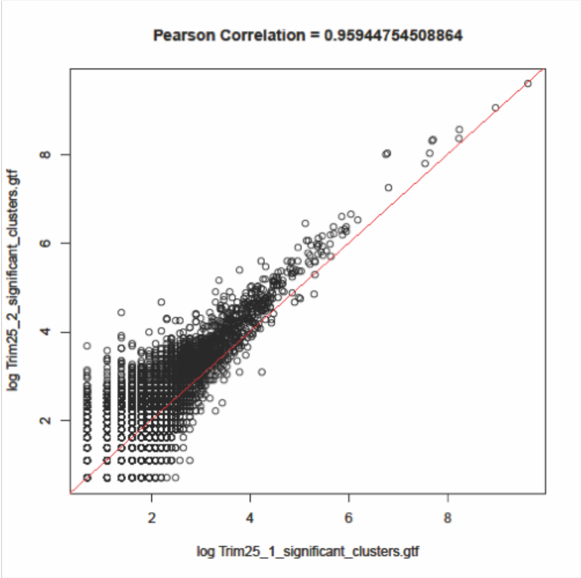
Pearson Correlation = 0.95944754508864
log Trim25_2_significant_clusters.gtf
log Trim25_1_significant_clusters.gtf

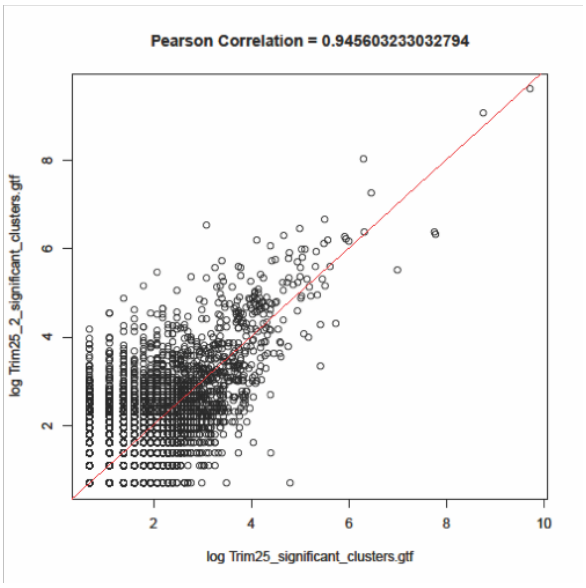
Pearson Correlation = 0.945603233032794
log Trim25_2_significant_clusters.gtf
log Trim25_significant_clusters.gtf

Supplementary Figure 4. TRIM25 CLIP-seq experiments are reproducible. The CLIP-seq of T7-TRIM25 was repeated three times. The first time with RNase at 1:1000 (TRIM25) and the two others at 1:10000 (TRIM25_1 and TRIM25_2). The circles represent merged clusters from each experiment. Pearson Correlation was calculated based on the height of overlapping clusters between experiments.

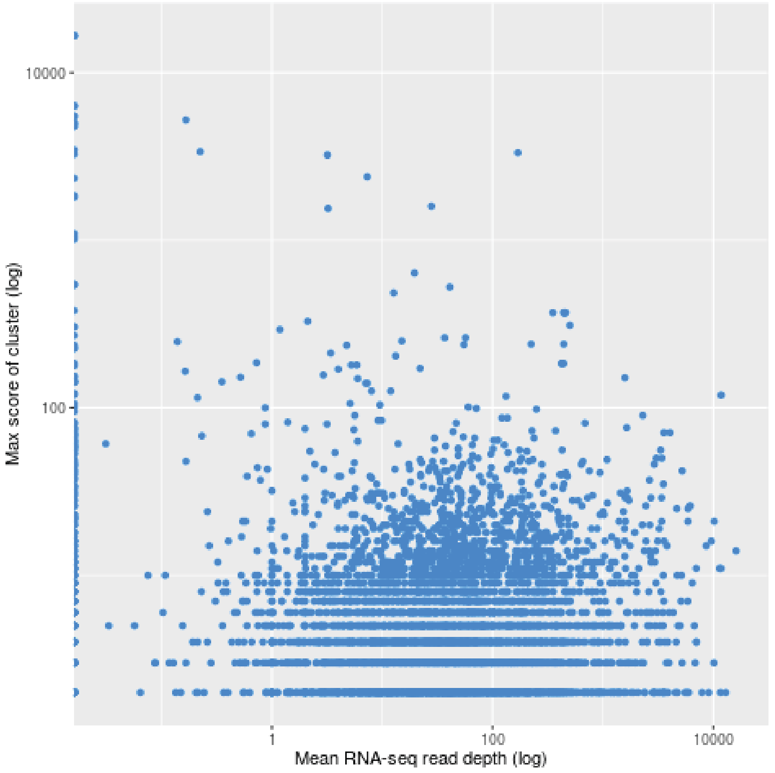

Supplementary Figure 5. There is no correlation of TRIM25 CLIP-seq cluster intensities with transcript abundance in HeLa cells. The heights of the clusters common in all three experiments were compared to the transcript abundance derived from RNA sequencing data for HeLa cells.

a

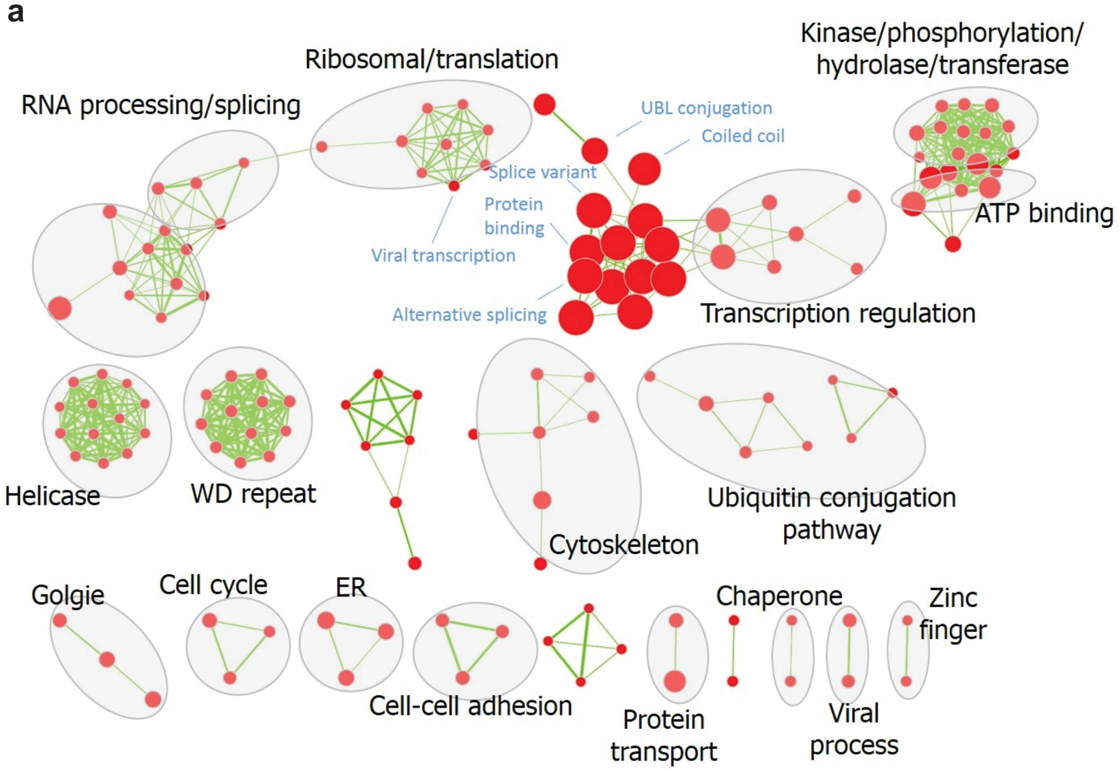
RNA processing/splicing
Ribosomal/translation
Kinase/phosphorylation/
hydrolase/transferase
UBL conjugation
Coiled coil
Splice variant
Protein
binding
Viral transcription
Alternative splicing
ATP binding
Transcription regulation
Helicase
WD repeat
Cytoskeleton
Ubiquitin conjugation
pathway
Golgie
Cell cycle
ER
Cell-cell adhesion
Protein
transport
Chaperone
Zinc
finger
Viral
process

b

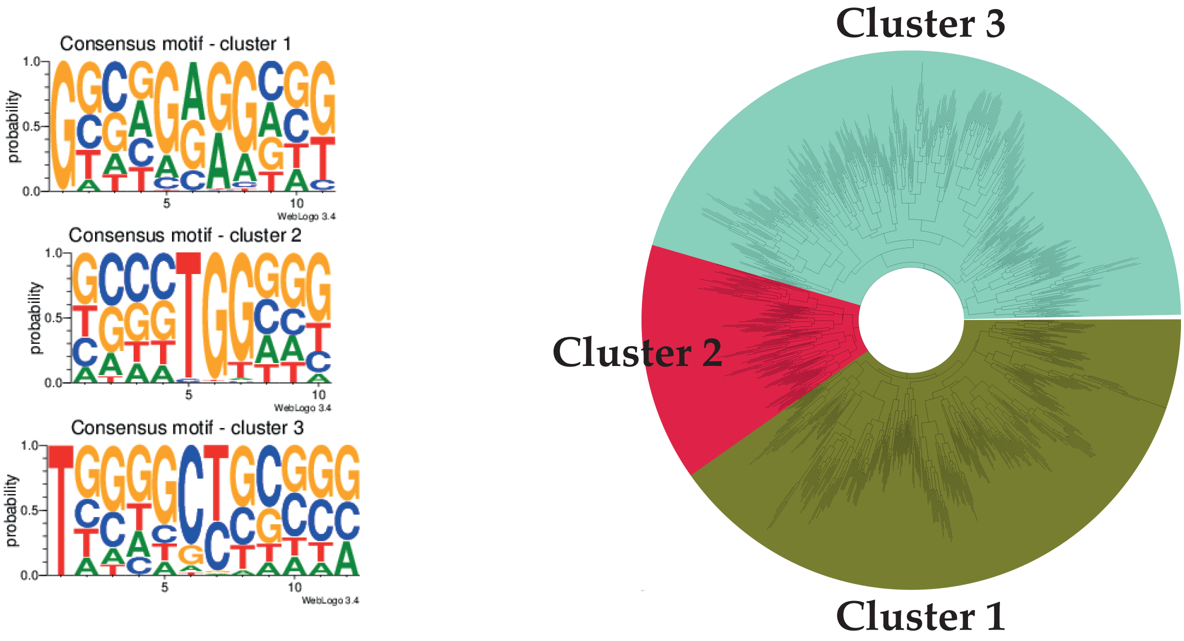
Consensus motif - cluster 1
Consensus motif - cluster 2
Consensus motif - cluster 3
probability
WebLogo 3.4
Cluster 3
Cluster 2
Cluster 1

Supplementary Figure 6. (a) GO-term annotation of TRIM25-bound transcripts from the CLIP-seq experiment shows several nodes including RNA processing and translation, WD repeats, phosphorylation and ubiquitination. GO-term annotations for enriched transcript was done using DAVID and visualized with the use of Cytoscape with the Enrichment Map Plugin. **(b)** The ab initio-derived TRIM25-binding binding sequences cluster in three motif clusters. T is representative of U in the RNA.

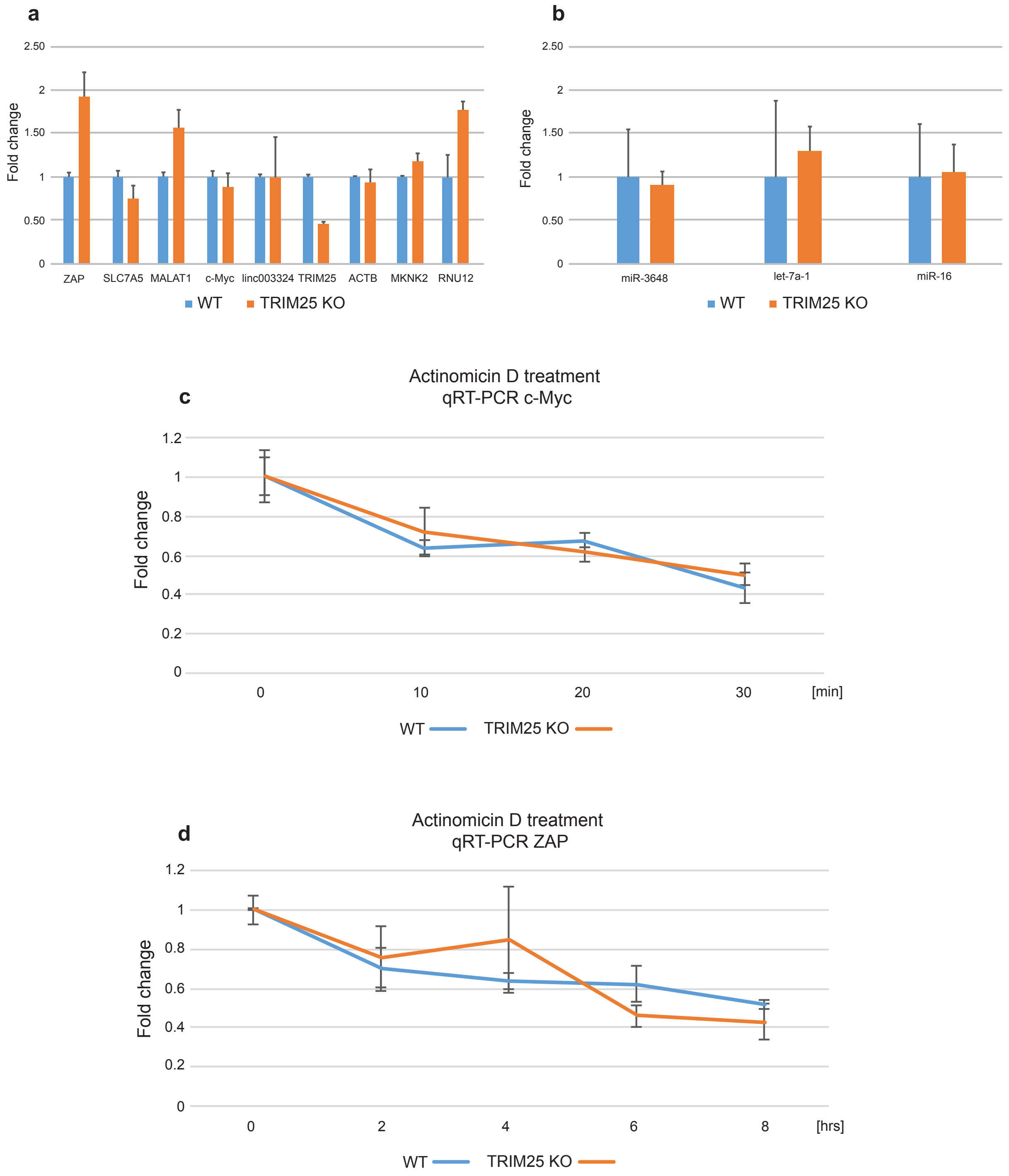

Supplementary Figure 7. TRIM25 does not influence RNA stability of selected targets. Real-time qRT-PCR of RNAs **(a)** and miRNAs **(b)** from HeLa wild type (WT – blue bars) and TRIM25 KO cells (orange bars). These RNA values were normalised to GAPDH whereas microRNA levels to 5S RNA. The fold change in the corresponding RNA abundance in the

TRIM25 KO cells was plotted relative to values from a WT control cell line, which were set to 1. Mean values and s.d. of three independent experiments are shown. Time-depended Actinomycin D treatment followed by qRT-PCR on c-Myc **(c)** and ZAP **(d)** mRNAs from WT (blue lines) and TRIM25 KO cells (orange lines) reveals no significant change in the RNA stability. The RNA values were normalised to 18S RNA. The fold change in the corresponding RNA abundance followed by Actinomycin D treatment was plotted relative to values obtained from the untreated control cells, which were set to 1. Mean values and s.d. of three independent experiments are shown.

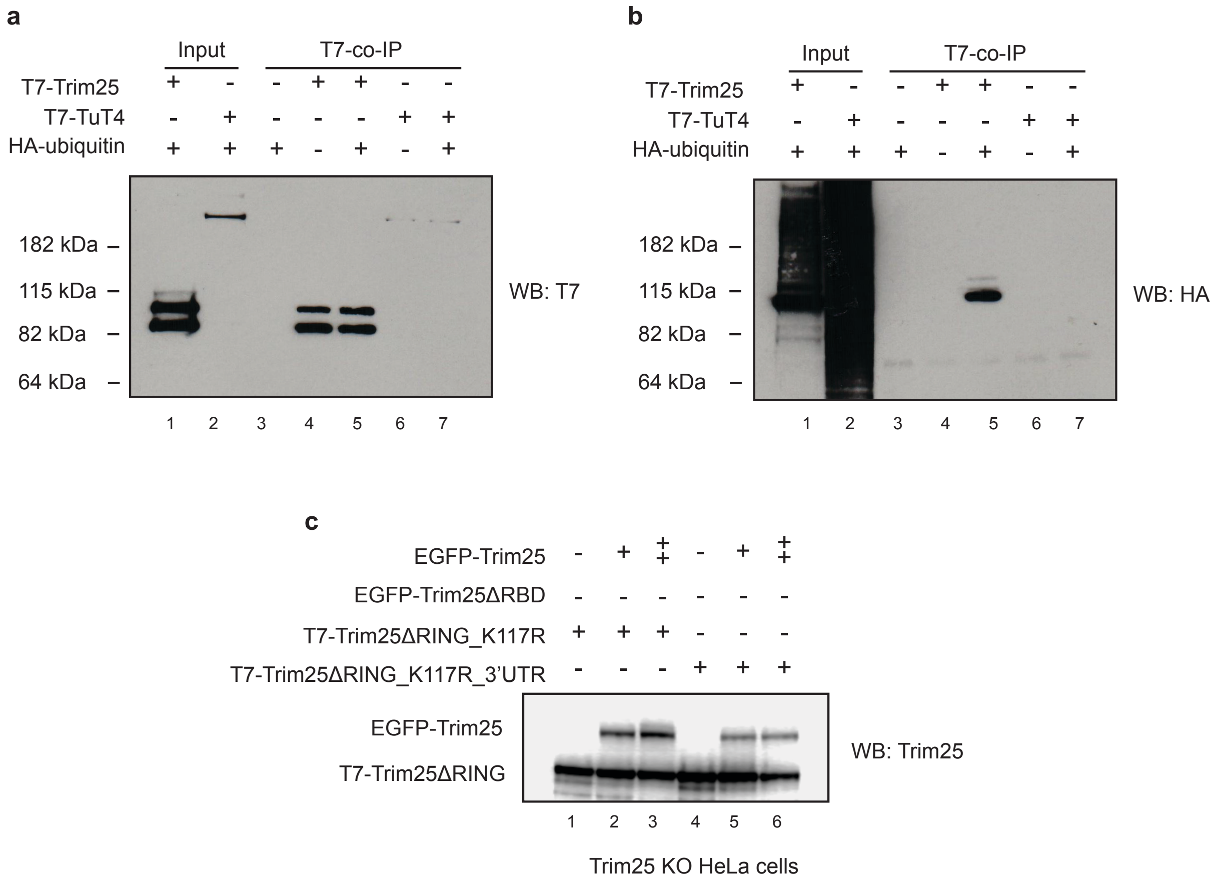

Supplementary Figure 8. TRIM25 undergoes ubiquitination. (a) T7-TRIM25 or T7-TUT4 was overexpressed together with HA-ubiquitin (400ng per well) after 24 hours the cells were treated with MG132 (10.5 μM) for 3 hours to block the proteasome and the proteins were immunoprecipitated with anti-T7 antibody. The bound proteins were separated on a SDS-PAGE gel and immunopreciptated proteins detected by anti-T7 antibody**. (b)** The ubiquitinated proteins were detected by anti-HA antibody. Lanes 1 and 2 show input T7-TRIM25 with HA-ubiquitin or T7-TUT4 with HA-ubiquitin respectively. Lanes 3 show overexpression of HA-ubiquitin only. Lanes 4 and 6 show T7-TRIM25 or T7-TUT4 respectively and 5 and 6 the same proteins co-expressed with HA-ubiquitin. **(c)** Western blot analysis of TRIM25 KO HeLa cells transfected with plasmids coding for T7-TRIM25ΔRING_K117R (250 ng) or T7-TRIM25ΔRING_K117R_3'UTR (500 ng) as well as increasing amounts of plasmids coding for EGFP-TRIM25 or EGFP-TRIM25ΔRBD. Lane 1 shows T7-TRIM25ΔRING_K117R control. Lanes 2 and 3 represent results of transfections with T7-TRIM25ΔRING_K117R and

increasing amounts of EGFP-TRIM25 (250ng and 500ng). Lane 4 shows T7-TRIM25ΔRING__K117R _3’UTR control. Lanes 5 and 6 represent results of transfections with T7-TRIM25ΔRING_K117R _3’UTR and increasing amounts of EGFP-TRIM25 (250ng and 500ng).
